# The plastidial retrograde signal methyl erythritol cyclopyrophosphate is a regulator of salicylic acid and jasmonic acid crosstalk

**DOI:** 10.1093/jxb/erv550

**Published:** 2016-01-04

**Authors:** Mark Lemos, Yanmei Xiao, Marta Bjornson, Jin-zheng Wang, Derrick Hicks, Amancio de Souza, Chang-Quan Wang, Panyu Yang, Shisong Ma, Savithramma Dinesh-Kumar, Katayoon Dehesh

**Affiliations:** ^1^Department of Plant Biology, University of California Davis, Davis, CA 95616, USA; ^2^Department of Plant Sciences, University of California Davis, Davis, CA 95616, USA; ^3^Genome Center, University of California, Davis, Davis, CA 95616, USA

**Keywords:** Coronatine-insensitive1 (COI1), hormonal interplay, jasmonic acid (JA), MEcPP (2-C-methyl-d-erythritol cyclopyrophosphate), plastidial retrograde signaling metabolite, salicylic acid (SA), stress responses.

## Abstract

The exquisite harmony between hormones and their corresponding signaling pathways is central to prioritizing plant responses to simultaneous and/or successive environmental trepidations. The crosstalk between jasmonic acid (JA) and salicylic acid (SA) is an established effective mechanism that optimizes and tailors plant adaptive responses. However, the underlying regulatory modules of this crosstalk are largely unknown. Global transcriptomic analyses of mutant plants (*ceh1*) with elevated levels of the stress-induced plastidial retrograde signaling metabolite 2-C-methyl-D-erythritol cyclopyrophosphate (MEcPP) revealed robustly induced JA marker genes, expected to be suppressed by the presence of constitutively high SA levels in the mutant background. Analyses of a range of genotypes with varying SA and MEcPP levels established the selective role of MEcPP-mediated signal(s) in induction of JA-responsive genes in the presence of elevated SA. Metabolic profiling revealed the presence of high levels of the JA precursor 12-oxo-phytodienoic acid (OPDA), but near wild type levels of JA in the *ceh1* mutant plants. Analyses of coronatine-insensitive 1 (*coi1*)/*ceh1* double mutant plants confirmed that the MEcPP-mediated induction is JA receptor COI1 dependent, potentially through elevated OPDA. These findings identify MEcPP as a previously unrecognized central regulatory module that induces JA-responsive genes in the presence of high SA, thereby staging a multifaceted plant response within the environmental context.

## Highlight

2-C-Methyl-D-erythritol cyclopyrophosphate is an isoprenoid intermediate and a dynamic plastidial stress-specific signal that calibrates salicylic acid–jasmonic acid crosstalk and induces jasmonic acid-responsive genes in the presence of high salicylic acid in a manner dependent on the F-box protein COI1.

## Introduction

To cope with hostile environmental conditions or attacks by pathogens or insects, plants have myriad intricately interrelated defense mechanisms, such as the biosynthesis of appropriate phytohormones and subsequent activation of signaling pathways tailored to the specific stress. Among the most intensively studied phytohormones known to play a pivotal role in the induction and regulation of adaptive responses against abiotic and/or biotic stresses are jasmonates and salicylic acid (SA).

Jasmonates, comprising jasmonic acid (JA) and derivatives, as well as the JA precursor 12-oxo-phytodienoic acid (OPDA), are a group of rapidly synthesized lipid-derived bioactive compounds produced via the oxylipin biosynthetic pathway in response to infection by necrotrophic pathogens, herbivores, or mechanical wounding (Gfeller *et al.*, 2010; Verhage *et al.*, 2010). Subsequent formation of the JA–isoleucine conjugate jasmonoyl-L-isoleucine (JA-Ile) followed by the binding of this endogenous active ligand to the F-box protein CORONATINE INSENSITIVE1 (COI1) leads to ubiquitination and consequent degradation of jasmonate zim (JAZ) repressor proteins by the 26S proteasome (Katsir *et al.*, 2008; Sheard *et al.*, 2010; Yan *et al.*, 2009). This degradation disrupts the physical interaction between JAZ proteins and transcriptional activators and results in derepression of the JA signaling pathway and subsequent activation of a large number of JA-responsive genes (Gonzalez-Cabanelas *et al.*, 2015; Kazan and Manners, 2008; Pieterse *et al.*, 2012; Thines *et al.*, 2007; Wasternack and Hause, 2013). The JA signaling pathway in *Arabidopsis thaliana* (Arabidopsis) is divided into two antagonistically controlled branches (Pieterse *et al.*, 2012; Pre *et al.*, 2008; Verhage *et al.*, 2011). The basic helix–loop–helix leucine zipper transcription factor MYC2, induced by insect herbivores, activates the MYC2-branch marker gene *vegetative storage protein 2* (*VSP2*) (Pieterse *et al.*, 2012; Pre *et al.*, 2008). The ethylene response factor 1 (ERF1) branch of the JA pathway, induced by necrotrophic pathogens, controls the expression of the ERF-branch marker gene *plant defensin 1.2* (*PDF1.2*). The gaseous phytohormone ethylene plays both a synergistic and inhibitory role in the JA pathway, in that it induces the ERF1 branch, while it antagonizes the MYC2 branch (Pre *et al.*, 2008). This antagonism between the two JA-pathway branches is further demonstrated by a previously reported induction of the MYC2 branch and suppression of the ERF1 branch after attack by herbivorous insects (Verhage *et al.*, 2011).

SA is a phenolic phytohormone typically involved in defense against biotrophic pathogens (Kunkel and Brooks, 2002; Pieterse *et al.*, 2012; Verhage *et al.*, 2010). The synthesis of SA is via the phenylalanine and/or isochorismate pathways (Garcion and Métraux, 2007), but in Arabidopsis the isochorismate pathway is favored (Ogawa *et al.*, 2007; Wildermuth *et al.*, 2001). Accumulation of SA results in the activation of a suite of biotic stress-responsive genes, including *pathogenesis-related1* (*PR1*) whose expression is often used as an SA signaling marker (Fu and Dong, 2013; Garcion and Métraux, 2007; Mou *et al.*, 2003; Pieterse *et al.*, 2012; Tada *et al.*, 2008).

The regulatory crosstalk of reciprocal antagonism between JA-dependent responses to insect herbivores or necrotrophs and SA-dependent responses to biotrophs is well documented (Doherty *et al.*, 1988; Gupta *et al.*, 2000; Koornneef *et al.*, 2008; Koornneef and Pieterse, 2008; Leon-Reyes *et al.*, 2010; Pieterse *et al.*, 2012; Spoel *et al.*, 2003; Thaler *et al.*, 2012). In Arabidopsis, the expression of the JA-response genes *PDF1.2* and *VSP2* is suppressed in the presence of elevated SA levels caused by pathogen infection or through exogenous application of SA (Koornneef *et al.*, 2008; Leon-Reyes *et al.*, 2009; Leon-Reyes *et al.*, 2010; Liu *et al.*, 2012; Pieterse *et al.*, 2012; Spoel *et al.*, 2003; Thaler *et al.*, 2012; Zander *et al.*, 2010). This antagonism is not limited to suppression of JA-dependent marker genes, but also encompasses the regulation of JA biosynthesis, as evidenced by suppression of JA accumulation in wounded tomato plants exogenously treated with SA or aspirin (Pena-Cortés *et al.*, 1993). Conversely, SA hydroxylase-expressing NahG plants unable to accumulate SA produced a 25-fold increase in JA levels and displayed enhanced expression of the JA-responsive genes, including *PDF1.2*, and *VSP2*, in response to infection by the SA-inducing pathogen *Pseudomonas syringae* (*Pst*) as compared with infected wild type Arabidopsis, which accumulates SA (Stintzi *et al.*, 2001).

Plant plastids function as both central metabolic hubs and environmental sensors that perceive stress and produce retrograde signals to coordinate nuclear-encoded adaptive responses. We have identified the plastid-derived metabolite 2-C-methyl-D-erythritol-2,4-cyclodiphosphate (MEcPP), a precursor of isoprenoids produced by the conserved and essential plastidial methylerythritol phosphate (MEP) pathway, as a critical stress-specific retrograde signaling metabolite that communicates plastidial perturbations to the nucleus in plants (Walley *et al.*, 2015; Wang *et al.*, 2015; Xiao *et al.*, 2012; Xiao *et al.*, 2013). This discovery was founded on a genetic screen that led to the isolation of a mutant line designated *ceh1*, for constitutive expression of *hydroperoxide lyase* (*HPL*), an otherwise stress-inducible nuclear gene encoding a plastidial enzyme in the HPL branch of the oxylipin pathway (Chehab *et al.*, 2006; Lorenzo *et al.*, 2003; Seemann *et al.*, 2005). The *ceh1* mutant is the result of a point mutation causing the substitution of leucine for phenylalanine in *(E)-4-hydroxy-3-methylbut-2-enyl-diphosphate synthase* (*HDS*), a nuclear gene encoding the plastidial enzyme responsible for the reduction of MEcPP to (E)-4-hydroxy-3-methyl-but-2-enyl pyrophosphate (HMBPP). This mutation results in the accumulation of MEcPP to high levels and consequent induction of selected stress-responsive nuclear genes and their respective metabolites (Xiao *et al.*, 2012). Among the nuclear genes with enhanced expression level in *ceh1* is *isochorismate synthase 1* (*ICS1*), a stress-inducible nuclear gene encoding a key plastidial enzyme in the SA biosynthetic pathway. Increased basal expression of *ICS1* in *ceh1* resulted in increased levels of SA, and by extension enhanced resistance to the biotrophic pathogen *Pst* strain DC3000 (Xiao *et al.*, 2012). The elevated SA levels in *ceh1* plants suggest that at least part of the MEcPP-mediated regulatory function is via SA phytohormone signaling.

Accumulation of MEcPP in response to a range of environmental perturbations (Li and Sharkey, 2013; Rivasseau *et al.*, 2009; Xiao *et al.*, 2012) raised the question of whether this stress-specific retrograde signal could, either directly or indirectly, regulate hormonal crosstalk and thus fine-tune plant stress responses. Specifically, the high SA levels in the *ceh1* mutant led us to examine SA–JA crosstalk using a combination of metabolic profiling and molecular genetic approaches. Here we report that a high level of SA in *ceh1* mutant fails to fully suppress JA pathway genes, and that the MEcPP-mediated activation of JA-pathway genes in *ceh1* mutant is dependent on JA receptor COI1. These findings establish a novel role for MEcPP in modulation of both SA and JA pathway genes and the suitability of *ceh1* mutant as an experimental platform for unravelling novel determinants of JA–SA crosstalk.

## Materials and methods

### Microarray analysis

Raw microarray data (.cel files) for *ceh1*, parent, and wild type plants (GSE61675) were analysed and processed into expression values using the gcRMA algorithm (Wu *et al.*, 2004). Genes up- or down-regulated ‘≥2-fold with *P*-value ≤0.05’ (two-tailed *t*-test) were identified as significantly altered genes in the *ceh1* mutant. The analyses were expanded to identify genes significantly altered by SA or JA (methyl jasmonate; MeJA) 3h after treatment using the publically available AtGenExpress data set (ME00364 and ME00337 from https://www.arabidopsis.org/portals/expression/microarray/ATGenExpress.jsp). Heatmaps were generated selecting significantly up-regulated genes from *ceh1* sorted according to their fold change and were compared with their corresponding expression values from SA- and JA-treated plant datasets.

### Plant growth and treatment

Plants used in this study include parent (*P*_*HPL*_::*LUC* reporter line) and previously described *ceh1*, *ceh1/eds16-1*, and *eds16-1* lines (Walley *et al.*, 2015; Xiao *et al.*, 2012); *ssi2* (SALK_036854), a T-DNA insertion in the second intron of *SSI2* obtained from TAIR; *mekk1-5*, a mutant described previously (Bjornson *et al.*, 2014); and the previously generated *coi1-1* mutant line (Xie *et al.*, 1998).

*Arabidopsis thaliana* plants were grown in a 16h light–8h dark cycle at 22 °C for 2 weeks on half-strength MS medium (Sigma-Aldrich M0404).

Exogenous application of 100 μM MeJA and 1mM SA individually or in combination in 0.015% Silwet L-77 or only Silwet L-77 as control was conducted by spraying 2-week-old plants 24h prior to tissue collection. All tissues were collected between 11.00h and 13.00h, flash frozen in liquid nitrogen, and stored at –80 °C until use.

### Expression analysis

Expression analysis was carried out by quantitative reverse transcription PCR (qRT-PCR) as previously described (Walley *et al.*, 2008). qRT-PCR was conducted in reaction mixture containing cDNA synthesized from total RNA, using iQ SYBR Green Supermix (Bio-Rad Laboratories), with appropriate primers (Supplementary Table S1 at *JXB* online). AT4G26410 was used as the internal standard for transcript normalization, as previously described (Walley *et al.*, 2008).

### MEcPP extraction and quantification using liquid chromatography and mass spectrometry

MEcPP was extracted by a slightly modified method previously described (Xiao *et al.*, 2012). Briefly, samples were analysed using a Thermo Finnigan Micro AS autosampler HPLC system coupled to a Thermo Fisher LTQ-Orbitrap XL mass spectrometer with an electrospray ionization source. Plant samples and standards were separated using an Accucore-150-Amide-HILIC column (150×2.1mm; particle size 2.6 µm; Thermo Scientific 16726-152130) with a guard column containing the same column matrix (Thermo Scientific 852-00; 16726-012105). The separation was conducted in isocratic conditions using 60% acetonitrile with 0.1% formic acid and 40% 50mM ammonium formate buffer pH 4.5. Flow rate was kept at 150 µl min^–1^ and the volume injected was 5 µl. The column was kept at room temperature. Mass spectra were acquired in negative ion mode under the following parameters: spray voltage, 4.5kV; sheath gas flow rate of 15 and capillary temperature of 275 °C. Samples were quantified using an external standard curve of MEcPP (Echelon, I-M054) with concentrations of 200, 100, 75, 60, 45, 36, 27, 13.5 and 6.75 µM, and final quantifications were normalized to starting fresh weight.

### Phytohormone quantification

Quantification of SA, JA and OPDA was carried out by gas chromatography–mass spectrometry (GC-MS), using dihydro-JA and deuterated SA and abscisic acid as internal standard, as previously described (Savchenko *et al.*, 2010).

## Results

### JA-responsive genes are induced in the *ceh1* mutant despite high SA levels

**Fig. 1.**
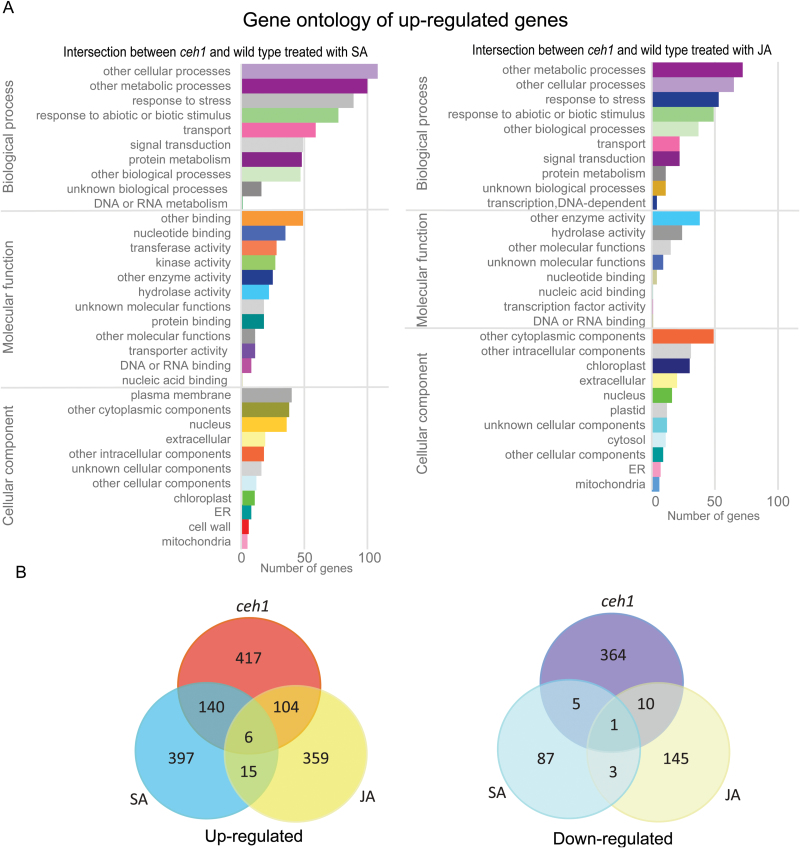
Comparative analysis of genes with modulated expression in *ceh1* mutant and wild type plants treated with SA or JA. (A) Gene ontology of up-regulated genes within the intersection of *ceh1* and SA-treated (left), and *ceh1* and JA-treated (right) wild type plants. Only significantly over-represented categories are shown as determined by Classification SuperViewer (http://bar.utoronto.ca/). (B) Venn diagram of up- or down- regulated transcripts (≥2-fold, *P*<0.05) in *ceh1* and wild type treated with SA or JA.

We performed global microarray analyses to examine the nature of genes whose transcript levels are robustly modulated in the *ceh1* mutant background (Walley *et al.*, 2015). The gene ontology (GO) term analyses not only identified a number of induced SA marker genes as predicted, but also surprisingly a significant number of JA marker genes expected to be suppressed by the constitutively high levels of SA in the *ceh1* mutant background (Fig. 1A and Supplementary Fig. S1). This finding prompted us to carry out comparative analyses between the microarray data for the *ceh1* mutant and those previously reported for the wild type Col-0 Arabidopsis plants exogenously treated with SA and JA (Fig. 1A, B). Surprisingly, in a three-way comparison we found only seven genes whose transcripts are robustly altered in both *ceh1* and wild type plants treated with SA and JA (Fig. 1B), thereby confirming the selectivity of these hormones in transcriptional regulation of genes that directly or indirectly tailor plant stress responses. In contrast, there is a notable overlap amongst genes with altered expression levels in *ceh1* compared with SA treatment and *ceh1* compared with JA treatment (Fig. 1A, B and Supplementary Fig. S1). This overlap is most prevalent amongst the induced rather than suppressed genes. Specifically, 140 SA-responsive genes, including SA-responsive gene *PR1* (AT2G14610), as well as 104 JA-responsive genes, including the marker gene *PDF1.2* (AT5G44420), are induced in the *ceh1* mutant background (Fig. 1A, B and Supplementary Fig. S1). The absence of greater overlap of genes between *ceh1* mutant and the exogenously SA-treated wild type plant is potentially due to the constitutive versus transient presence of SA in the *ceh1* and wild type plants, respectively. However, while induction of the SA-responsive genes as the result of constitutively high SA levels in *ceh1* (Xiao *et al.*, 2012) is fully expected, the induction of JA marker genes in the mutant is unanticipated.

**Fig. 2.**
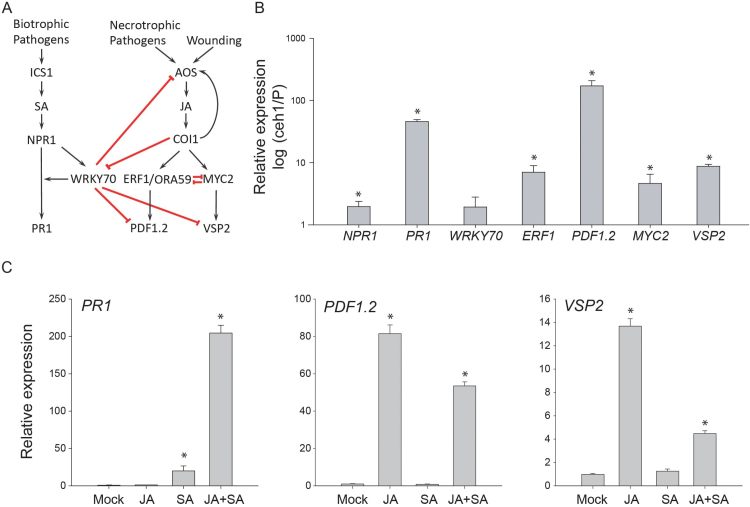
High SA does not impede induction of JA-responsive genes in the *ceh1* mutant. (A) Schematic representation of SA and JA pathway genes and their interactions. (B) Relative expression levels of SA and JA marker genes in *ceh1* compared with parent line (*P*_*HPL*_ *::LUC*). Data are means of three biological replicates and three technical replicates ±SEM. Asterisks denote significant differences determined by Student’s *t* test (*P*<0.05). (C) Relative expression of genes with altered transcript levels in mock, JA-, SA-, and JA+SA-treated wild type Col-0 plants. Data are means of three biological replicates and three technical replicates ±SEM. Asterisks denote significant differences determined by Student’s *t* test (*P*<0.05).

To validate the microarray data, we compared the expression levels of a subset of SA- and JA-responsive genes in the Col-0 parent line expressing *P*_*HPL*_ *::LUC* and the *ceh1* mutant plants (Fig. 2A). The expression level of a key regulator of the SA response pathway gene, *NPR1*, is modestly but significantly higher in *ceh1* compared with parent line (Fig. 2A). Moreover, expression of *PR1*, the gene downstream of *NPR1*, is also notably higher in *ceh1* than that of the parent plant (Fig. 2A, B). In agreement with the microarray data, concomitant with increased SA and SA-dependent gene transcripts, the expression levels of the JA-responsive genes from both the wound-induced MYC2 branch and the necrotrophic pathogen-induced ERF1 branch are also elevated in *ceh1* relative to the parent plant (Fig. 2A, B). However, the expression levels of genes in the ERF1 branch are altered more markedly than those of the MYC2 branch. Specifically, the transcript levels of both *ERF1* and its target gene *PDF1.2* are higher in *ceh1* as compared with parent line (Fig. 2A, B).

The simultaneous induction of the genes within the MYC2 and ERF1 branches of the JA pathway in *ceh1* suggests that the previously reported antagonistic control of MYC2 and ERF1/ORA59 over the two branches (Verhage *et al.*, 2010; Verhage *et al.*, 2011) is at least partly abolished in *ceh1* mutant.

Next, we examined the expression level of *WRKY70*, a convergence node between JA- and SA-dependent pathways by virtue of activating SA-induced genes and repressing JA-responsive genes (Li *et al.*, 2004; Li *et al.*, 2006). Interestingly, RT-qPCR analyses confirmed the previously published microarray data in *ceh1* mutant plants (Walley *et al.*, 2015), establishing that *WRKY70* transcript level is not significantly altered between *ceh1* and parent line (Fig. 2A, B). These data collectively indicate that *WRKY70* may not play a principal role in modulating SA–JA crosstalk in the *ceh1* mutant. This result is in contrast with the recent report showing enhanced levels of *WRKY70* in a *ceh1* mutant allele named *hds3* (Gonzalez-Cabanelas *et al.*, 2015). It is possible that different mutation sites within the HDS enzyme between *ceh1* and *hds3* could contribute to an accumulation of different levels of MEcPP leading to differential potency of the signal. Alternatively, it could be due to variation in experimental approaches. Exogenous application of SA has also been shown to activate *WRKY70* expression (Li *et al.*, 2004). The unaltered *WRKY70* transcript levels in *ceh1* as compared with parent line could stem from MEcPP interception of the SA-mediated induction of *WRKY70.*

The difference between our findings using the *ceh1* mutant and the established antagonistic effects of high SA on the expression levels of JA-responsive genes in the wild type background (Koornneef *et al.*, 2008; Leon-Reyes *et al.*, 2009; Leon-Reyes *et al.*, 2010; Pieterse *et al.*, 2012; Spoel *et al.*, 2003; Zander *et al.*, 2010) led us to examine the contribution of our experimental conditions. Thus, we examined the expression levels of JA and SA marker genes in the wild type plants exogenously treated with SA and JA, either individually or in combination, under the same experimental conditions employed for *ceh1* mutant lines. In accordance with the published results, the combined application of SA+JA as compared with JA alone notably reduced the expression of the JA-specific markers *PDF1.2* and *VSP2* (Fig. 2C). Interestingly and in agreement with the previous report (Leon-Reyes *et al.*, 2010), the combined application of SA and JA amplified the expression of the canonical SA marker gene *PR1* well above the levels observed with SA alone (Fig. 2C).

Collectively, these data validate the authenticity of failure of high SA levels in suppressing expression of JA-responsive genes in the *ceh1* mutant background, and demonstrate the predicted SA-mediated suppression of JA-response genes in the wild type background under the experimental conditions employed.

### Constitutively high SA levels fail to repress levels of JA precursor 12-OPDA in the *ceh1* mutant

The marked difference in transcript levels of JA marker genes in the *ceh1* mutant versus SA-treated plants led us to test the differential effects of constitutively elevated SA in *ceh1* plants compared with transiently heightened SA levels in suppression of jasmonates and JA marker genes. To test this, we used *suppressor of SA insensitivity2* (*ssi2*) and *mitogen activated protein kinase kinase kinase1*-5 (*mekk1-5*) mutants with constitutively elevated SA (Bjornson *et al.*, 2014; Kachroo *et al.*, 2001; Shah *et al.*, 2001). In addition, to discriminate between the potential role of high MEcPP from constitutively elevated SA levels in induction of JAs and the respective marker genes, we also employed an SA-deficient mutant, *enhanced disease susceptibility 16-1* (*eds16-1*), encoding a dysfunctional *isochorismate synthase 1* (*ICS1*) (Wildermuth *et al.*, 2001), together with the *ceh1*/*eds16-1* double mutant that contains high MEcPP but is deficient in SA (Xiao *et al.*, 2012).

**Fig. 3.**
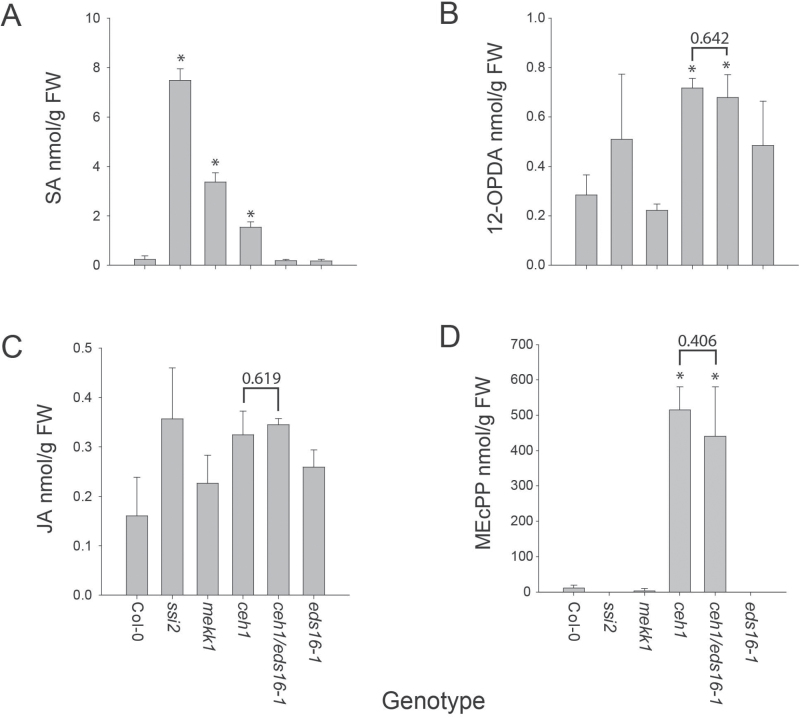
Constitutively high SA levels fail to repress levels of JA precursor 12-OPDA in the *ceh1* mutant. Analyses of the levels of SA (A), 12-OPDA (B), JA (C) and MEcPP (D) in Col-0, *ssi2*, *mekk1-5*, *ceh1*, *ceh1/eds16-1*, and *eds16-1* genotypes. Data are means of three biological replicates ±SD. Asterisks denote significant differences from Col-0 as determined by Student’s *t* test (*P*<0.05). Brackets and above-indicated *P* value denote significance or the lack of between *ceh1* and *ceh1/eds16-1* as determined by Student’s *t* test.

Hormonal profiling of these various mutant genotypes under our experimental conditions clearly shows hierarchical levels of SA, with the highest levels present in *ssi2* followed by *mekk1-5* and then *ceh1* (Fig. 3A). As expected, almost equally negligible levels of SA were detected in control Col-0, *eds16-1*, and *eds16-1*/*ceh1* mutant plants (Fig. 3A).

In contrast to SA level, the JA basal level is not significantly different amongst these various mutants and wild type Col-0, indicating lack of adverse effects of SA on JA accumulation. Interestingly, levels of the JA precursor 12-OPDA are moderately but significantly and equally higher in *ceh1* and *ceh1/eds16-1* as compared with the other genotypes (Fig. 3B, C).

Since MEcPP activates the stress-responsive SA biosynthesis gene *ICS1* leading to accumulation of SA (Xiao *et al.*, 2012), we questioned the possible reciprocity of high SA resulting in accumulation of MEcPP. Metabolic profiling of MEcPP in *ssi2* and *mekk1-5*, the mutants with constitutively high SA, showed similar or below detection levels of MEcPP compared with wild type Col-0, while *ceh1* and *ceh1/eds16-1* displayed similarly highly elevated levels of MEcPP compared with wild type (Fig. 3D).

Together these findings provide evidence for an SA-independent accumulation of MEcPP, and additionally support an SA-independent but MEcPP-dependent induction of OPDA in *ceh1* and *ceh1*/*eds16-1*. The distinct SA and MEcPP signatures among different mutants described here position us to differentiate between their individual signaling roles in SA–JA crosstalk.

### MEcPP-mediated induction of JA marker genes are COI1 dependent

**Fig. 4.**
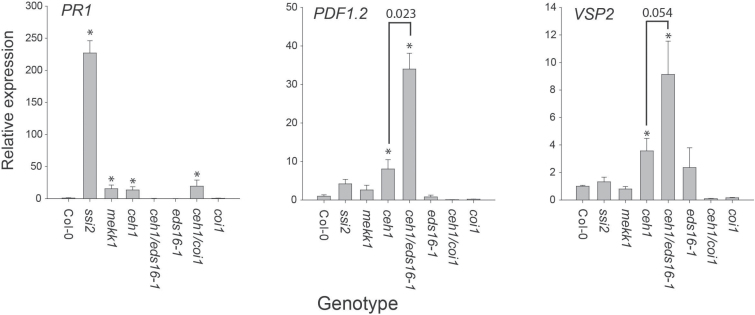
MEcPP interference with SA suppression of JA marker genes is COI1 dependent. Relative expression levels of *PR1*, *PDF1.2*, and *VSP2* in Col-0, *ssi2*, *mekk1-5*, *ceh1*, *ceh1/eds16-1*, *ceh1/coi1*, *coi1*, and *eds16-1* genotypes. Data are means of three biological replicates and three technical replicates ±SEM. Asterisks denote significant differences from Col-0 as determined by Student’s *t*-test (*P*<0.05). Brackets and above-indicated *P* value denote significance or the lack of between *ceh1* and *ceh1/eds16-1* as determined by Student’s *t*-test.

To gain insight into the underlying mechanism involved in SA- versus MEcPP-mediated signaling, we performed gene expression analysis of the SA- and JA-dependent marker genes *PR1*, *PDF1.2* and *VSP2* among different mutants with high SA and control genotypes. The level of *PR1* expression correlated well with SA levels, with *ssi2* displaying the highest *PR1* transcript levels followed by *mekk1-5* and *ceh1*, and near wild type levels among the other genotypes (Fig. 4).

Transcript levels of the ERF1-branch JA marker gene, *PDF1.2*, were increased 8- and over 35-fold in *ceh1* and *ceh1/eds16-1*, respectively, as compared with wild type (Fig. 4). The differential expression level of *PDF1.2* in *ceh1* versus *ceh1/eds16-1* strongly supports a role of MEcPP-mediated signaling in mitigating SA suppression of JA marker genes. Moreover, comparable expression levels of *PDF1.2* in *ssi2*, *mekk1-5*, and wild type plants (Fig. 4) support the notion that the activation of *PDF1.2* in *ceh1* is not due to the presence of constitutively high SA. Similarly, the MYC2 branch of the JA-dependent marker gene *VSP2* is induced in *ceh1* and *ceh1/eds16-1* and not in *mekk1-5* or *ssi2.* These results further support a MEcPP-dependent but SA-independent induction of this JA-responsive gene (Fig. 4).

The enhanced levels of 12-OPDA (Fig. 3B), in conjunction with the established function of OPDA in modulating gene expression via COI1 in a manner distinct from JA (Ribot *et al.*, 2008), prompted us to examine the role of COI1 in induction of the JA-responsive genes in the *ceh1* mutant. Therefore, we generated the *ceh1*/*coi1* double mutant using the previously generated *coi1-1* mutant line (Xie *et al.*, 1998), which for simplicity here is referred to as *coi1*. Next, the transcript levels of *PR1*, *PDF1.2* and *VSP2* were examined in wild type, *ceh1*, *ceh1/coi1* and *coi1* mutant genotypes (Fig. 4). These results clearly show similar *PR1* expression levels in *ceh1* and *ceh1*/*coi1* mutant, indicating COI1-independent induction of this gene. In contrast, while basal levels of *PDF1.2* and *VSP2* are enhanced in *ceh1* as compared with wild type control, the levels are highly diminished in *coi1* and *ceh1*/*coi1*double mutant plants. These results indicate that MEcPP-mediated induction of JA-marker genes requires COI1.

## Discussion

The exquisite harmony between hormones and their respective signaling cascades is central to optimizing virtually all metabolic and physiological aspects of plant adaptation to environmental perturbations. The interplay between JA and SA is one optimizing strategy employed by plants to prioritize and tailor responses to the nature of the attack encountered. However, under natural conditions plants are challenged not by individual enemies, but rather by simultaneous or sequential attacks by myriad adversaries. As such, plants have evolved an integrated signaling cascade to fine-tune tailored responses rapidly and appropriately to biotic challenges within the context of the abiotic perturbations of the prevailing environment.

MEcPP is a precursor of isoprenoids produced by the plastidial MEP pathway, which also functions as a retrograde plastid-to-nucleus signaling metabolite as well as an interorgannellar communication signal modulating the expression levels of selected stress-response genes (Walley *et al.*, 2015; Wang *et al.*, 2015; Xiao *et al.*, 2012; Xiao *et al.*, 2013). Consistent with the stress-specific signaling role of MEcPP, many environmental stresses increase the levels of this dynamic metabolite (Ge *et al.*, 2012; Li and Sharkey, 2013; Mongelard *et al.*, 2011; Rivasseau *et al.*, 2009; Xiao *et al.*, 2012). The induction of MEcPP levels by a wide range of stresses, combined with an induction of SA- and JA-response genes in the high MEcPP-containing *ceh1* mutant background prompted us to investigate the role of this signaling metabolite in the fine-tuning of SA–JA antagonism. A combination of exogenous application of hormones to wild type plants and utilization of various mutants with increased endogenous levels of SA and MEcPP, both individually and in combination, established SA-independent MEcPP-mediated induction of JA-responsive genes. However, stronger induction of JA marker genes in the SA-deficient *ceh1*/*eds16-1* mutant line as compared with *ceh1* is a clear indication of the inability of MEcPP to fully mitigate the SA-mediated suppression of JA marker gene expression. The data presented here clearly illustrate a direct or indirect role for MEcPP in fine-tuning SA–JA antagonism, thereby enabling plants to respond effectively to multiple and simultaneous challenges encountered. Moreover, basal levels of JA in all genotypes examined suggest either that induction of JA-responsive genes in high MEcPP-containing genotypes is independent of JA levels, or alternatively, that higher MEcPP levels may have led to a JA hypersensitivity response.

Interestingly, however, high MEcPP-containing mutants display statistically significant increases in the levels of 12-OPDA as compared with genotypes with basal MEcPP levels. The accumulation of the precursor rather than the final product, JA, potentially implies that translocation of 12-OPDA from the chloroplast to the peroxisome, the site of β-oxidation for JA production, might be compromised in *ceh1* plants. Alternatively, the β-oxidation pathway might function inefficiently in the high MEcPP-containing *ceh1* mutant. Regardless, higher levels of expression of *PDF1.2* and *VSP2* might be mediated by 12-OPDA. This is an active signal molecule that up-regulates COI1-dependent genes that are also regulated by JA, and is also capable of inducing in a COI1-independent fashion genes that are not induced by JA, as well as regulating the expression of genes in a COI1-dependent fashion albeit independently of JA (Ribot *et al.*, 2008; Stintzi *et al.*, 2001; Taki *et al.*, 2005). Examining the *ceh1*, *ceh1/coi1*, and *coi1* genotypes clearly enabled us to show that induction of the JA marker genes *PDF1.2* and *VSP2* is via a COI1-dependent pathway.

Our studies illustrate the absence of antagonism between MYC2 and ERF1 and their corresponding marker genes in *ceh1*, thus suggesting that high MEcPP intercepts the previously noted negative crosstalk between these two branches of JA signaling (Pre *et al.*, 2008; Verhage *et al.*, 2011). This, together with activation of JA-response genes in the presence of high SA levels, expands the role of MEcPP to a signaling component that reorganizes and tweaks hormonal input in plant stress responses.

Collectively, data presented here provide a better understanding of the interconnected complex networks constituting an exquisitely measured regulatory mechanism fine tuning plant adaptive stress responses.

**Fig. 5.**
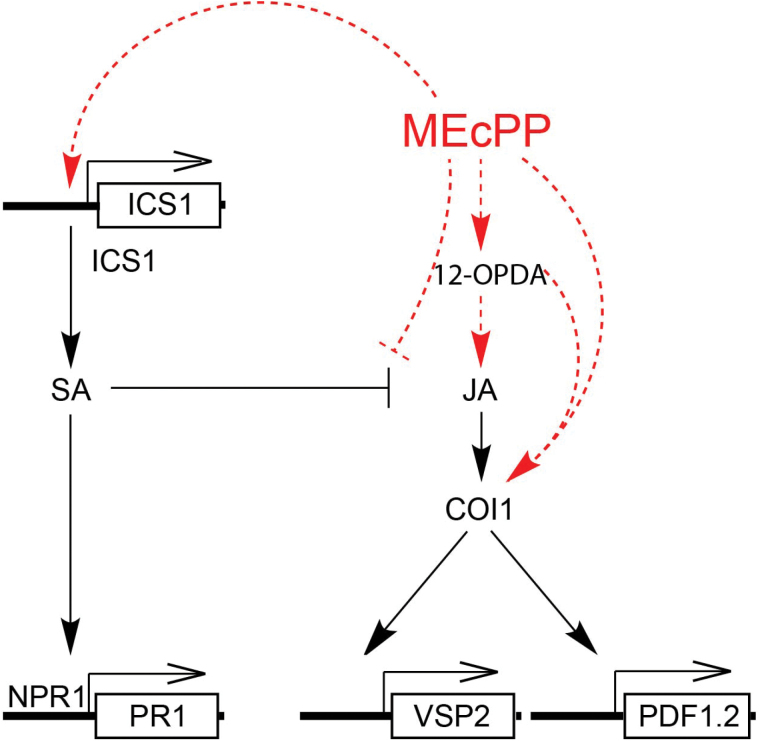
Schematic model of MEcPP calibrating SA–JA antagonism via a COI1-dependent but JA-independent path. Stress specific accumulation of MEcPP either through increased levels of 12-OPDA or directly via a COI1-dependent but JA-independent path induces of *VSP2* and *PDF1.2*, thereby calibrating the SA-mediated suppression of JA-responsive genes.

Our finding supports a model (Fig. 5) in which MEcPP mediates induction of the known JA marker genes through 12-OPDA and COI1 in an SA-independent manner. This finding adds another layer of regulatory complexity to the flow of information between the plastids and nucleus critical in plant adaptive responses to environmental stresses. Future assembly of these data into functional modules will provide insight into a more unified model of the retrograde stress response network that controls stress response pathways.

## Supplementary data

Supplementary data are available at *JXB* online.

Fig. S1. Intersection of genes between *ceh1* and JA-treated wild type plants.

Table S1. Primer list.

Supplementary Data
