# Supplementary material for: The plastidial retrograde signal methyl erythritol cyclopyrophosphate is a regulator of salicylic acid and jasmonic acid crosstalk
Source: J Exp Bot. 2016 Jan 4;67(5):1557–66. doi: 10.1093/jxb/erv550 (PMC4762391; doi:10.1093/jxb/erv550)
Supplement: Supplementary Data [file supp_67_5_1557__index.html]

The plastidial retrograde signal methyl erythritol cyclopyrophosphate is a regulator of salicylic acid and jasmonic acid crosstalk — The plastidial retrograde signal methyl erythritol cyclopyrophosphate is a regulator of salicylic acid and jasmonic acid crosstalk — Supplementary Data 

# The plastidial retrograde signal methyl erythritol cyclopyrophosphate is a regulator of salicylic acid and jasmonic acid crosstalk

## Supplementary Data

Data files

- Supplementary\_Figure\_S1.jpg - Supplementary Data
- supplementary\_table\_S1.pdf - Supplementary Data
