# Supplementary figures and images for: The plastidial retrograde signal methyl erythritol cyclopyrophosphate is a regulator of salicylic acid and jasmonic acid crosstalk
Source: J Exp Bot. 2016 Jan 4;67(5):1557–66. doi: 10.1093/jxb/erv550 (PMC4762391; doi:10.1093/jxb/erv550)

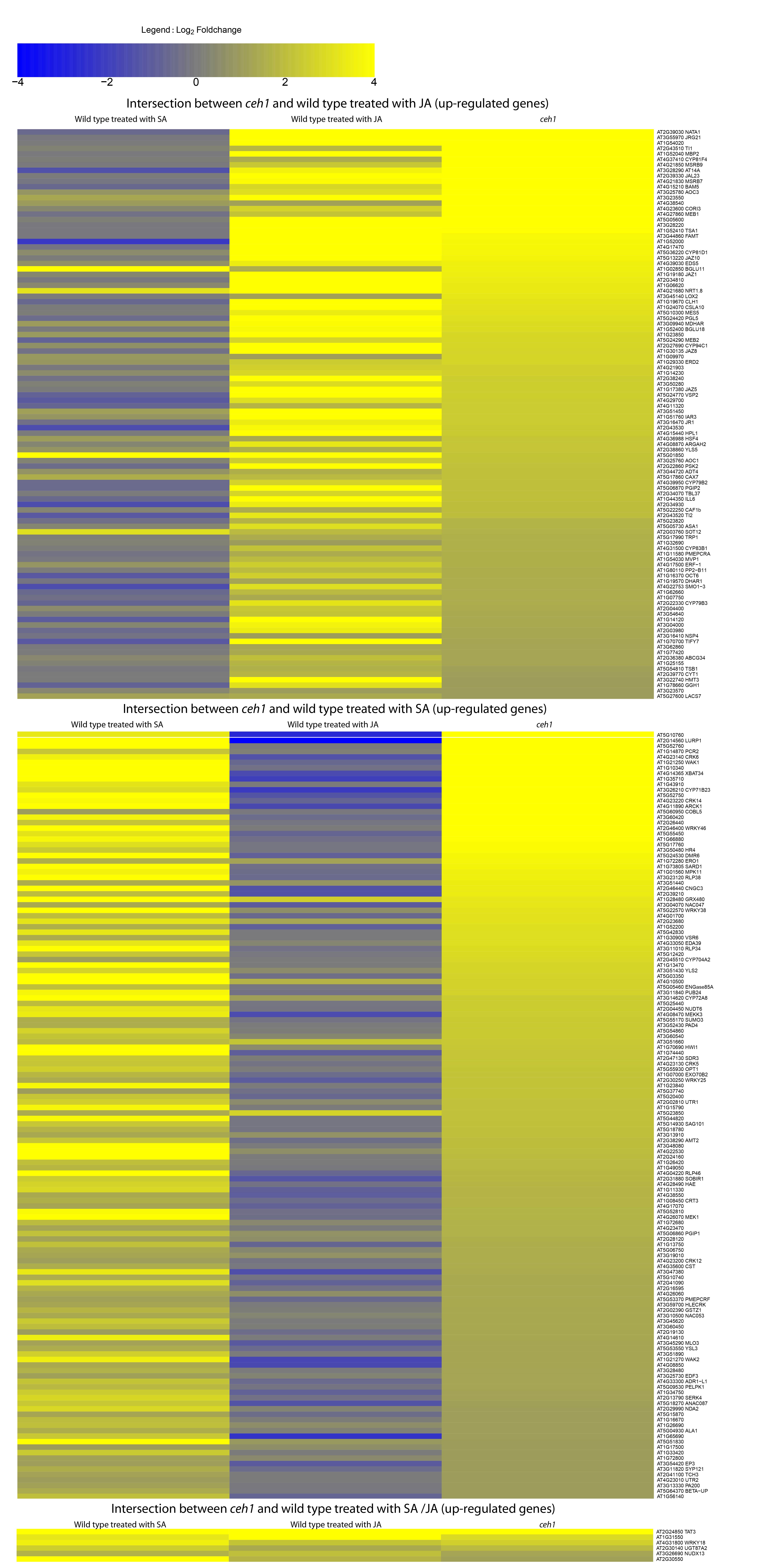

Supplement: Supplementary Data [file supp_erv550_Supplementary_Figure_S1.jpg]
