# Supplementary material for: The plastidial retrograde signal methyl erythritol cyclopyrophosphate is a regulator of salicylic acid and jasmonic acid crosstalk
Source: J Exp Bot. 2016 Jan 4;67(5):1557–66. doi: 10.1093/jxb/erv550 (PMC4762391; doi:10.1093/jxb/erv550)
Supplement: Supplementary Data [file supp_erv550_supplementary_table_S1.pdf]

Journal of Experimental Botany

The plastidial retrograde signal MEcPP is a regulator of SA and JA crosstalk

Mark Lemos, Yanmei Xiao, Marta Bjornson, Jin-zheng Wang, Derrick Hicks, Amancio de Souza, Chang-Quan Wang, Panyu Yang, Shisong Ma, Savithramma Dinesh-Kumar, and Katayoon Dehesh

Supplementary Table S1. List of qRT-PCR primers.

Gene	Forward Primer 5'→3' sequence	Reverse Primer 5'→3' sequence
NPR1	TGTTCCTCCCTCTTTTGCAG	GATCTCCATTGCAGCTTGTG
PR1	GTGGGTTAGCGAGAAGGCTA	ACTTTGGCACATCCGAGTCT
WRKY70	AACACCAACGCAGAAACTCC	CGAACCATGATGACGATGAG
MYC2	TCATACGACGGTTGCCAGAA	AGCAACGTTTACAAGCTTTGATTG
PDF1.2	TTTGCTGCTTTCGACGCAC	CGCAAACCCCTGACCATG
VSP2	TCAGTGACCGTTGGAAGTTGTG	GTTCGAACCATTAGGCTTCAATATG
ERF1	TTCCCTTCAACGAGAACGAC	TAGGTTTGTTGCGTGGACTG
AT4G26410	GAGCTGAAGTGGCTTCCATGAC	GGTCCGACATACCCATGATCC
